# Pharmacodynamic Effect of Clopidogrel in Patients Undergoing Transcatheter Aortic Valve Implantation

**DOI:** 10.1155/2013/386074

**Published:** 2013-07-15

**Authors:** Petr Tousek, Viktor Kocka, Jakub Sulzenko, Frantisek Bednar, Hana Linkova, Petr Widimsky

**Affiliations:** Third Faculty of Medicine, Charles University, University Hospital Vinohrady, Cardiocenter, Srobarova 50, 100 34 Prague 10, Czech Republic

## Abstract

The aim of this study was to analyze periprocedural and mid-term effect of clopidogrel on platelet function using the VerifyNow P2Y_12_ point-of-care assay in patients undergoing TAVI. Platelet reactivity was measured at the beginning of the procedure after 300 mg clopidogrel bolus administration and during the follow-up (at 1 month after the procedure) in 52 patients undergoing TAVI using the Medtronic CoreValve prosthesis (Medtronic CoreValve). A cutoff value of 240 PRU was used to identify nonresponders to clopidogrel treatment with high residual platelet reactivity (HRPR). Baseline HRPR was identified in 80% of patients and in 72% of patients during 6-month follow-up. There was no significant difference in the pharmacodynamic effects of clopidogrel on platelet reactivity from baseline to 6-months follow-up (297 ± 57 vs. 275 ± 62; *P* = 0.058). Ischemic event occurred only in 3 patients (5.8%) from the study group. In conclusion, majority of patients undergoing TAVI had high residual platelet reactivity after pretreatment with 300 mg of clopidogrel and during the 6-month follow-up at dual antiplatelet treatment. The noneffectiveness of clopidogrel in the TAVI population raises the question of the routine use of dual antiplatelet treatment in this setting.

## 1. Introduction

The number of patients with severe aortic stenosis treated with transcatheter aortic valve implantation (TAVI) is increasing worldwide. This procedure is technically feasible in the majority of patients at high risk for standard surgical management. However, special attention should be given to potential periprocedural and short-term follow-up complications. Risks associated with TAVI differ and include ischemic (stroke, myocardial infarction) or bleeding (access site complication, cardiac perforation) complications at the same time. A loading dose of 300 mg of clopidogrel before TAVI followed by dual antiaggregation therapy with 75 mg of clopidogrel for 3–6 months is generally used. This therapeutic strategy significantly decreases ischemic events after percutaneous coronary intervention (PCI) [1]. The effect of clopidogrel on platelet reactivity can be tested and high residual platelet reactivity in approximately 30% of patients after PCI is associated with increased risk of cardiovascular death and myocardial infarction during follow-up [2, 3]. However, no data have been reported regarding the effectiveness of clopidogrel in the TAVI setting or its possible impact on adverse events. Therefore we analyzed the effect of clopidogrel on platelet function using the VerifyNow P2Y_12_ point-of-care assay in patients undergoing TAVI.

## 2. Methods

We did the analysis in patients with severe aortic stenosis who underwent TAVI using the Medtronic CoreValve prosthesis (Medtronic CoreValve) between April 2009 and July 2012. All interventions were performed after pretreatment with 300 mg of clopidogrel >12 h before the procedure. Dual antiplatelet therapy with 75 mg of clopidogrel and 100 mg of acetylsalicylic acid was recommended for 6 months followed by acetylsalicylic acid alone. Platelet reactivity after clopidogrel treatment was measured by the VerifyNow P2Y_12_ point-of-care assay (Accumetrics, San Diego, CA) at baseline (at the beginning of the procedure) and during the follow-up (at 1 moth after the procedure). The results were expressed as P2Y_12_ reaction units (PRU). A cutoff value of 240 PRU was used to identify non-responders to clopidogrel treatment with high residual platelet reactivity [2]. Continuous variables are expressed as mean ± standard deviation; nominal variables are expressed as count and percentages. Continuous variables were compared using the unpaired Student's *t*-test. A *P* value of *P* < 0.05 was considered significant.

## 3. Results

In total, 70 patients (average age 81 ± 6 years) underwent TAVI using the Medtronic CoreValve prosthesis. Baseline VerifyNow testing was successfully performed in 52 patients. In 8 patients dual antiplatelet treatment was not started due to the need of chronic oral anticoagulation and in 10 patients errors occurred during testing and thus platelet reactivity could not be assessed. Clinical characteristics of patients with baseline platelet reactivity testing are shown in Table 1. The procedure was successful in 51 patients. There were six major access site complications caused by major bleeding that required transfusion and were treated either surgically (in 3 cases) or by a conservative approach. In one patient, pericardiocentesis was performed for tamponade. Ischemic events occurred in three patients, of which one MI and one stroke occurred during the procedure and one patient suffered from stroke after discharge. One patient died during hospitalization for multiple organ failure. Mortality at 6 months was 3.8%. Baseline high residual platelet reactivity (HRPR) with PRU≥240 was identified in 42 (80%) patients. Average baseline PRU was 297 ± 57. Control VerifyNow testing was performed in 47 patients during 6-month follow-up. Thirty-four (72%) patients were assigned as nonresponsive with HRPR. Follow-up average PRU was 275 ± 62. There was no significant difference in the pharmacodynamic effects of clopidogrel on platelet reactivity from baseline to 6-months follow-up (*P* = 0.058).

## 4. Discussion

A strategy of clopidogrel pretreatment followed by long-term therapy is beneficial in reducing major cardiovascular events in patients undergoing PCI [4]. Major bleeding complications in these patients differ depending on antiaggregant used and do not exceed the rate of ischemic complications [5, 6]. This is not the case for TAVI where ischemic events are much less frequent than bleeding complications. Even though cerebral imaging studies have shown a very high incidence (66%–86%) of new ischemic defects after TAVI, most defects are clinically silent [7]. The incidence of clinically apparent stroke after TAVI has an average of about 3% [7]. A German multicenter registry including 697 patients treated with TAVI indicated rates of stroke and myocardial infarction of 2.8% and 0.3%, respectively [8]. These values were comparable with FRANCE-2 registry and those reported by Piazza and by Rodés-Cabau [9–11]. On the other hand, major access site bleeding complication is the most frequent procedural complication and is present in 5%–10% of patients undergoing TAVI [7, 9–11]. Thus, special attention should be paid to reducing these procedural bleeding events. One possible issue seems to be the strategy of clopidogrel administration. As shown in our observation, the rate of ischemic events remains acceptable even if the majority of patients has HRPR at the time of procedure and follow-up. This raises the question of whether it would be beneficial to use an individual approach for clopidogrel administration (e.g., previous PCI, diffuse coronary artery disease, previous stroke, etc.). Furthermore, our results can also explain findings of two small randomized study, where the strategy of adding clopidogrel to aspirin after TAVI was not superior to aspirin alone in the six-month patients outcomes [12, 13]. Atrial fibrillation is another important comorbidity that plays role in antithrombotic strategy management. Chronic atrial fibrillation occurs approximately in one-third of patient undergoing TAVI [7]. Several antithrombotic treatment regiments after TAVI are thus under investigations in ongoing trials [13] because the optimal treatment management is still not known.

## 5. Conclusion

In conclusion, we found that the majority of patients undergoing TAVI had high residual platelet reactivity after pretreatment with 300 mg of clopidogrel and during the 6-month follow-up at dual antiplatelet treatment. The noneffectiveness of clopidogrel in the TAVI population raises the question of the routine use of dual antiplatelet treatment in this setting. It is important to evaluate the antiplatelet treatment strategy in patients undergoing TAVI in the large randomized study.

## Figures and Tables

**Table 1:** Baseline characteristics of the study population (*n* = 52).

Age, years (SD)	81 (7)
Female, *n* (%)	29 (56)
BMI, kg/m^2^ (SD)	26.8 (5)
Logistic EuroScore, (SD)	23 (12)
Coronary artery disease, *n* (%)	28 (54)
eGFR <60 mL/min, *n* (%)	27 (52)
Atrial fibrillation/flutter, *n* (%)	9 (17)
Previous PCI, *n* (%)	10 (19)
Previous CABG, *n* (%)	11 (21)
Previous AVR with bioprosthesis,* n* (%)	1 (3)
LVEF, % (SD)	51 (12)
LVEF < 40%	17 (32)
AVA index, cm^2^/m^2^ (SD)	0.42 (0.08)
Femoral approach, *n* (%)	47 (90)
Subclavian approach, *n* (%)	5 (10)
